# Leukocyte-Specific Morrbid Promotes Leukocyte Differentiation and Atherogenesis

**DOI:** 10.34133/research.0187

**Published:** 2023-07-06

**Authors:** Di Xiang, Lei Jiang, Qiong Yuan, Yang Yu, Ruiming Liu, Meiting Chen, Zheng Kuai, Wendy Zhang, Fan Yang, Tingting Wu, Zhiyu He, Zuhui Ke, Wanzi Hong, Pengcheng He, Ning Tan, Yeying Sun, Zhen Shi, Xuebiao Wei, Jianfang Luo, Xiaoqiu Tan, Yuqing Huo, Gangjian Qin, Chunxiang Zhang

**Affiliations:** ^1^ Department of Cardiology, Key Laboratory of Medical Electrophysiology, Ministry of Education, Institute of Cardiovascular Research, The Affiliated Hospital of Southwest Medical University, Southwest Medical University, Luzhou, Sichuan 646000, China.; ^2^ Department of Biomedical Engineering, School of Medicine, The University of Alabama at Birmingham, Birmingham, AL 35233, USA.; ^3^ Department of Cardiology, Guangdong Cardiovascular Institute, Guangdong Provincial Key Laboratory of Coronary Heart Disease Prevention, Guangdong Provincial Institute of Geriatric Medicine, Guangdong General Hospital, Guangdong Academy of Medical Sciences, Guangzhou, Guangdong 510100, China.; ^4^ Vascular Biology Center, Medical College of Georgia, Augusta University, Augusta, GA 30912, USA.

## Abstract

Monocyte-to-M0/M1 macrophage differentiation with unclear molecular mechanisms is a pivotal cellular event in many cardiovascular diseases including atherosclerosis. Long non-coding RNAs (lncRNAs) are a group of protein expression regulators; however, the roles of monocyte-lncRNAs in macrophage differentiation and its related vascular diseases are still unclear. The study aims to investigate whether the novel leukocyte-specific lncRNA Morrbid could regulate macrophage differentiation and atherogenesis. We identified that Morrbid was increased in monocytes and arterial walls from atherosclerotic mouse and from patients with atherosclerosis. In cultured monocytes, Morrbid expression was markedly increased during monocyte to M0 macrophage differentiation with an additional increase during M0 macrophage-to-M1 macrophage differentiation. The differentiation stimuli-induced monocyte–macrophage differentiation and the macrophage activity were inhibited by Morrbid knockdown. Moreover, overexpression of Morrbid alone was sufficient to elicit the monocyte–macrophage differentiation. The role of Morrbid in monocyte–macrophage differentiation was also identified in vivo in atherosclerotic mice and was verified in Morrbid knockout mice. We identified that PI3-kinase/Akt was involved in the up-regulation of Morrbid expression, whereas s100a10 was involved in Morrbid-mediated effect on macrophage differentiation. To provide a proof of concept of Morrbid in pathogenesis of monocyte/macrophage-related vascular disease, we applied an acute atherosclerosis model in mice. The results revealed that overexpression of Morrbid enhanced but monocyte/macrophage-specific Morrbid knockout inhibited the monocytes/macrophages recruitment and atherosclerotic lesion formation in mice. The results suggest that Morrbid is a novel biomarker and a modulator of monocyte–macrophage phenotypes, which is involved in atherogenesis.

## Introduction

Phenotypic change from monocytes into macrophages is a pivotal cellular event in the development, homeostasis, tissue repair, and immune defense [1,2]. Disruption of the normal monocyte–macrophage differentiation could lead to diverse disorders such as inflammatory diseases, cardiovascular diseases, metabolic disorders, and tumors. Atherosclerosis is an inflammatory disease, where monocyte/macrophage activation and differentiation are central to the initiation and progression of atherosclerotic lesions [3–5]. Indeed, monocyte activation, infiltration into arterial wall, followed by differentiation into macrophage (M0) and inflammatory macrophage (M1) are key cellular events in atherogenesis [3–5]. However, the molecular mechanisms that govern monocyte/macrophage differentiation and functions remain largely unknown, which highlights the importance and urgency to discover novel molecular mechanisms of monocyte/macrophage differentiation and functions and develop new therapies for atherosclerosis [6].

Long non-coding RNAs (lncRNAs) are a group of protein expression regulators at the epigenetic level [7–10]. Recent studies have clearly demonstrated that lncRNAs have strong biological functions and are involved in the controls of many cellular functions and diverse disease processes including cardiovascular diseases [11,12]; however, to date, the roles of leukocyte-specific/enriched lncRNAs in the differentiation of monocyte into macrophage and in the development of inflammation-related vascular diseases are still an unexposed research field.

Morrbid (Gm14005) is a novel leukocyte-specific lncRNA identified in 2016 [13]. This monocyte-enriched lncRNA is located in Chromosome 2 and is conserved between mouse and human. In this *Nature* article, Kotzin et al. [13] reported that Morrbid is a critical gene in the lifespan control of the leukocytes (myeloid cells). The potential biological roles of this leukocyte-specific lncRNA in monocyte–macrophage differentiation and in leukocyte-related inflammatory vascular diseases are currently unknown. Our initial preliminary data have revealed that Morrbid expression is significantly increased during the monocyte–macrophage differentiation, and in monocytes from mice and patients with the enhanced monocyte–macrophage differentiation. We thus hypothesized that the leukocyte-enriched lncRNA-Morrbid may play important roles in phenotypic change from monocytes to macrophages and in leukocyte phenotype-related vascular diseases such as atherosclerosis via its target genes. The current study is to test our hypothesis at the molecular, cellular, animal, and patient levels.

## Results

### Morrbid expression is increased in monocytes from patients with the enhanced monocyte–macrophage differentiation

**Fig. 1.**
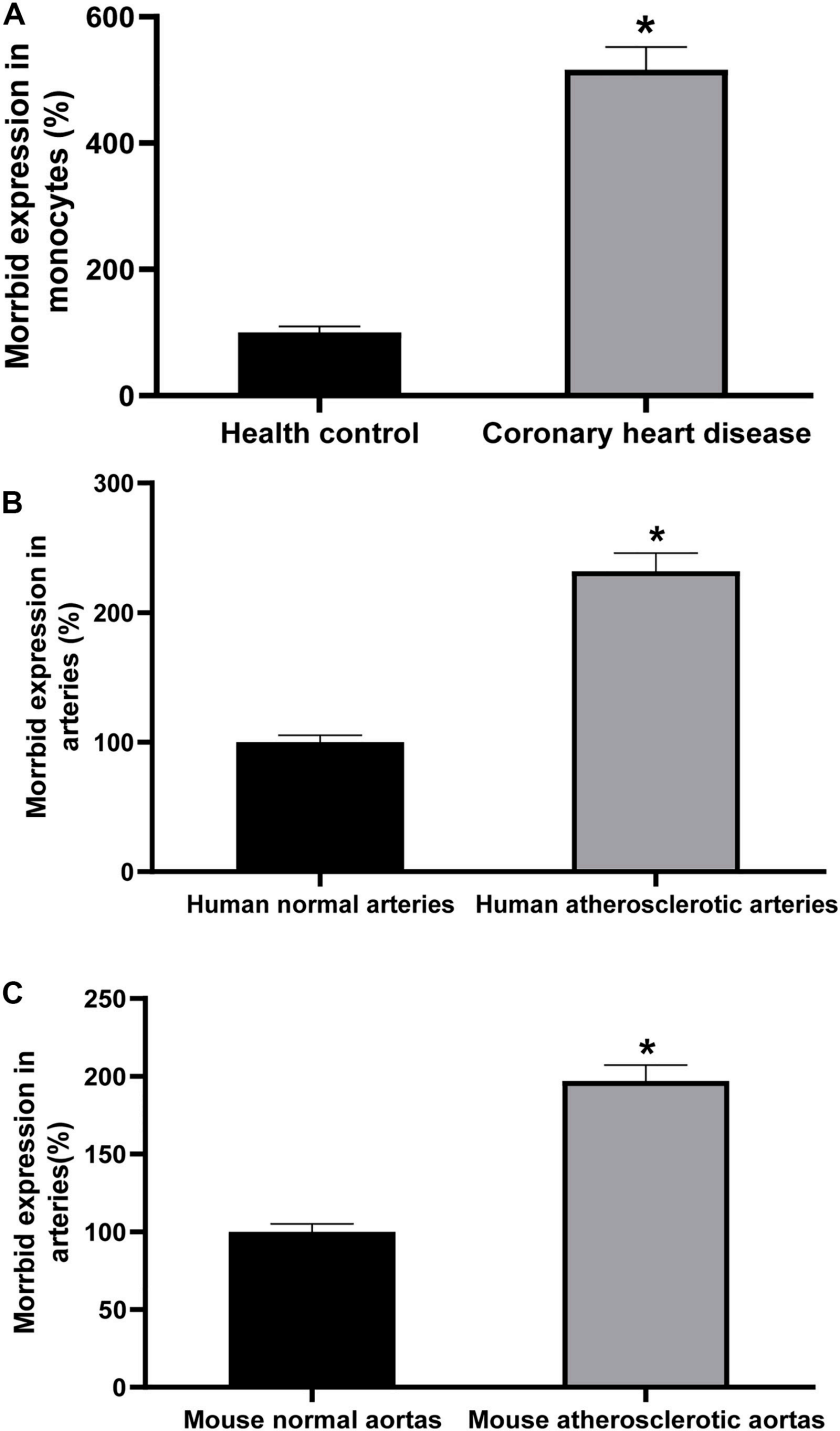
Morrbid expression is increased in monocytes and arteries from patients and mice with atherosclerotic disease. Morrbid expression in monocytes is increased from patients with coronary artery disease (CAD) (A). Morrbid expression is increased in human atherosclerotic lower limb arteries (B) and in mouse atherosclerotic aortas (C). The expression of Morrbid in these cells was determined by RT-PCR. Human circulating monocytes were isolated from 30 patients with CAD and from 30 age- and sex-matched healthy controls in (A). Note: *n* = 30; *P < 0.05 compared with the control group; human lower limb arteries were obtained from 6 patients with arteriosclerosis obliterans and 6 donors, and the 6 normal lower limb arteries from donors in (B). Note: *n* = 6; *P < 0.05 compared with the control group. Mouse 12 normal and 12 atherosclerotic aortas were from normal mice and ApoE-knockout mice with atherosclerosis induced by a Western diet in (C). Note: *n* = 12; *P < 0.05 compared with the control group.

It is well-established that patients with atherosclerotic vascular disease such as coronary artery disease (CAD) have the enhanced monocyte–macrophage differentiation. To determine the potential role of Morrbid in monocyte–macrophage differentiation, we measured the expression levels of Morrbid in circulating monocytes of patients with CAD and of normal control subjects. CAD patients (*n* = 30) were recruited from Guangdong General Hospital. The age- and sex-matched healthy controls (*n* = 30) were from the Health Examination Center of the same hospital. As shown in Fig. 1A, Morrbid expression in monocytes from CAD patients was higher than that from the healthy controls.

To test whether or not the increased Morrbid expression is a general phenomenon in monocytes with the enhanced monocyte–macrophage differentiation, we also determined its expression in circulating monocytes from patients (*n* = 12) with sepsis (endotoxemia), another well-known inflammatory disease with the increased monocyte–macrophage differentiation. The result showed that Morrbid expression level in monocytes from patients with sepsis was also higher than that from the age- and sex-matched healthy controls (*n* = 12) (Fig. S1). The results indicate that the increased expression of Morrbid was not disease-specific. Instead, it might be a general phenomenon in monocytes with the enhanced monocyte–macrophage differentiation under many disease conditions.

### Morrbid expression is increased in human and mouse atherosclerotic arteries

Atherosclerotic arterial walls have the increased infiltration of monocytes with the enhanced monocyte–macrophage differentiation. To explore the direct involvement of atherosclerotic lesion formation in vascular walls, we determined the levels of Morrbid in normal and atherosclerotic human lower limb arteries (Fig. 1B), and in normal and atherosclerotic mouse aortas (Fig. 1C). We found that Morrbid expression was increased in human and mouse atherosclerotic arteries [14], compared with that in their normal control arteries (Fig. 1B and C).

### Morrbid expression is up-regulated during monocyte-to-M0 macrophage differentiation with an additional increase during M0 macrophage-to-M1 macrophage differentiation

**Fig. 2.**
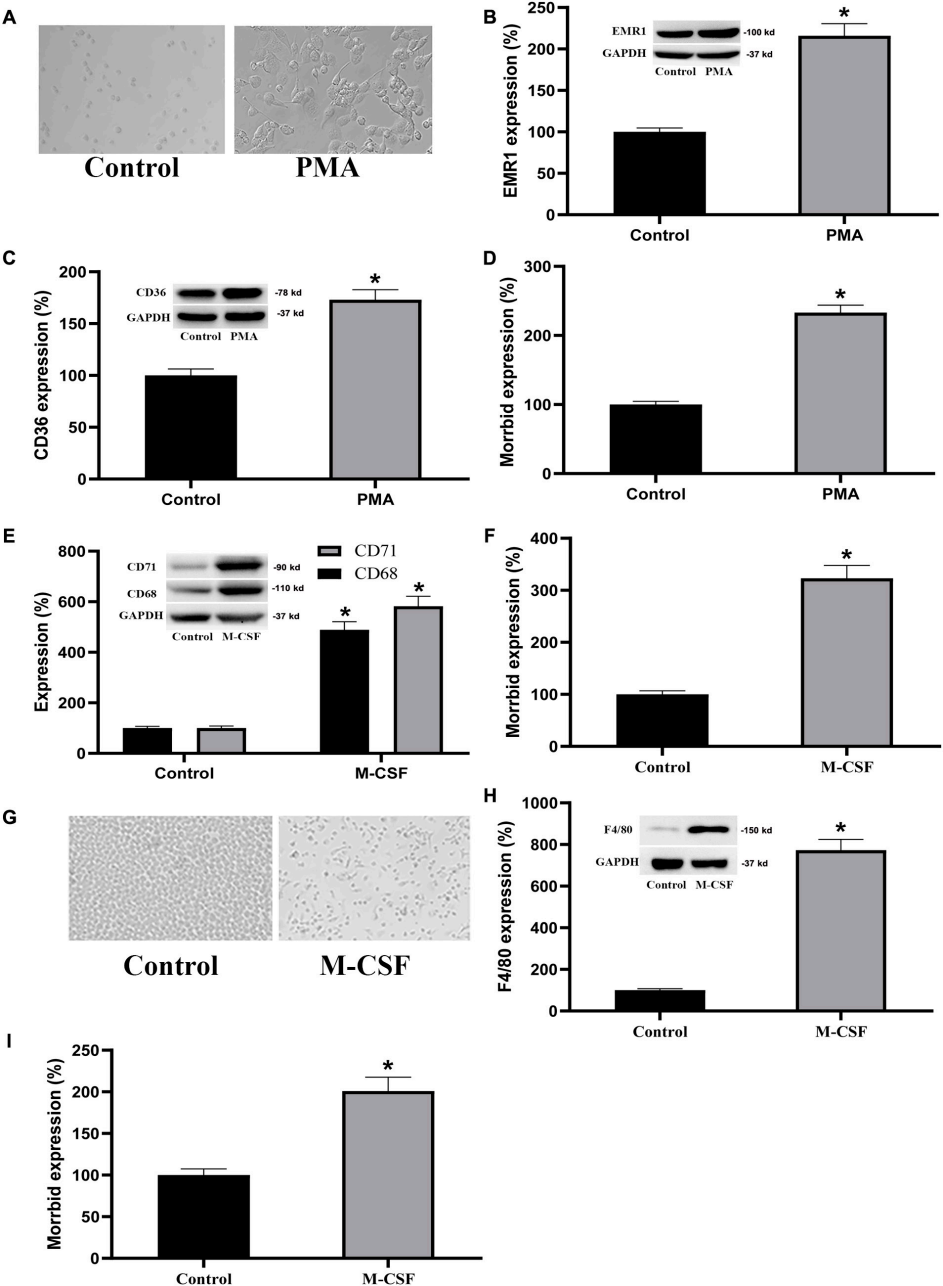
Morrbid expression is increased during monocyte–macrophage differentiation in human THP-1 monocytes, human primary monocytes, and mouse primary monocytes. THP-1 monocytes were cultured in PMA (100 nM) for 48 h, then analyzed for monocyte–macrophage differentiation by the increase in cell size (A) and expression of differentiation-associated maker genes, epidermal growth factor module-containing mucin-like receptor 1 (EMR1) (B) and CD36 (C), and for Morrbid gene expression (D). Human peripheral blood (huPB) monocyte–macrophage differentiation was induced by treatment with macrophage colony-stimulating factor (M-CSF) (50 ng/ml) for 48 h, then evaluated by increased macrophage differentiation maker genes, CD71 and CD68 (E), and Morrbid expression (F). Mouse bone marrow (BM)-derived monocyte–macrophage differentiation was induced by treatment with M-CSF (20 ng/ml) for 48 h, then assessed by increased cell size and adherence (G), increased expression of the mouse macrophage differentiation marker gene F4/80 (H), and Morrbid expression (I). *n* = 6; *P < 0.05 vs. controls.

To test the potential involvement of Morrbid in the differentiation from monocyte into macrophage, we first determined the expression changes of Morrbid during monocyte–macrophage differentiation using the human Tohoku Hospital Pediatrics-1 (THP-1) cell line. In this cell model, human THP-1 monocytes were differentiated into M0 macrophages by 48-h incubation with 100 nM phorbol myristate acetate (PMA) followed by 24-h incubation in Royal Park Memorial Institute (RPMI) medium [15]. As shown in Fig. 2A, monocyte–macrophage differentiation was successfully induced as shown by morphological changes (spreading on culture), adherence on culture dishes, and the increased expression of macrophage markers including EMR1 (epidermal growth factor module-containing mucin-like receptor 1) (Fig. 2B) and CD36 (Fig. 2C) [4]. Interestingly, the expression of Morrbid in THP-1 monocytes was significantly increased during their differentiation into macrophages (M0) (Fig. 2D).

Colony-stimulating factor-induced differentiation of peripheral blood mononuclear cells (PBMCs) is a well-accepted in vitro model for studying the monocyte–macrophage differentiation process in human primary cells. To verify the expression changes of Morrbid during macrophage differentiation in human primary cells, PBMCs isolated from healthy subjects were applied. The cultured PBMCs in RPMI-1640 medium were treated with either the macrophage colony-stimulating factor (M-CSF) (50 ng/ml) or vehicle for 48 h. The macrophage differentiation of human PBMCs was indeed induced as shown by the increased expression of CD71 and CD68 (Fig. 2E), the 2 macrophage differentiation markers of human PBMCs [4,16]. We found that the expression of Morrbid in PBMCs was significantly increased after they differentiated into macrophages (Fig. 2F).

To provide the association of Morrbid with monocyte–macrophage differentiation in other species, and to provide an evidence for our in vivo intervention studies, we determined the expression changes of mouse primary cells during monocyte–macrophage differentiation. In this experiment, mouse monocytes were obtained from mouse bone marrows as described [16,17]. Mouse monocyte–macrophage differentiation was induced by treatment with M-CSF (20 ng/ml) for 48 h as shown by morphological changes, the enhanced adherence, and the increased expression of mouse macrophage differentiation marker F4/80 [15] (Fig. 2G and H). Remarkably, the expression of Morrbid in mouse monocytes was increased after they differentiated into macrophages (Fig. 2I).

To induce M1 macrophages, THP-1-derived M0 macrophages were incubated with IFN-γ (20 ng/ml) and LPS (10 pg/ml) for 24 h. M1 macrophages were verified by the increased expression of M1 markers including tumor necrosis factor-α (TNFα), interleukin-6 (IL-6), and C-X-C Motif Chemokine 10 (CXCL10). Macrophage M2 polarization was induced by incubation of M0 macrophages with interleukin 4 (IL-4, 20 ng/ml) and IL-13 (20 ng/ml). The M2 phenotype was confirmed by the increased expression of M2 markers including CD206, CD163, IL-10, and CCL22. An additional up-regulation of Morrbid expression during M0 macrophage to inflammatory M1 macrophage differentiation was consistently observed (Fig. S2), but no difference in Morrbid expression was found between M0 and M2 groups. To further verify the change of Morrbid expression from M0 macrophages to M1 macrophages, we applied another Macrophage M1 polarization model in which M1 macrophages were induced by oxidized low-density lipoprotein (oxLDL), another pro-atherogenic stimulus [18]. The result demonstrated that the expression of Morrbid was increased after macrophage M1 polarization induced by oxLDL (80 μg/ml) treatment for 24 h (Fig. S3).

### Morrbid is a critical gene in monocyte–macrophage differentiation of cultured human and mouse cells in vitro

**Fig. 3.**
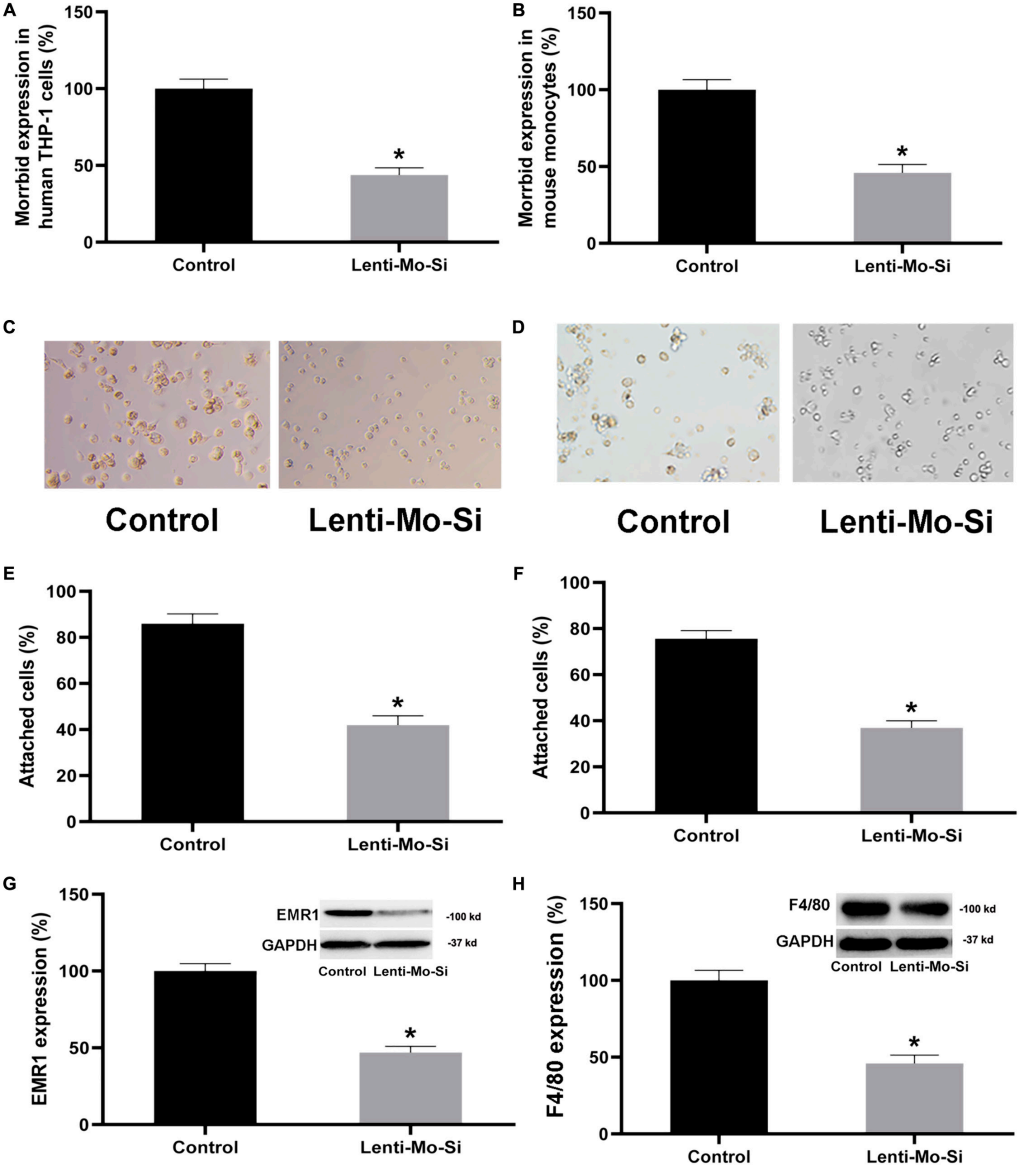
Knockdown of Morrbid attenuates monocyte–macrophage differentiation. Lenti-Mo-Si (20 MOI) was successfully transduced in human and mouse monocytes as shown by the decreased Morrbid expression (A and B). Monocyte–macrophage differentiation was induced by PMA 100 nmol/L for 48 h in human THP-1 monocytes (C, E, and G) or by M-CSF 20 ng/ml for 48 h in mouse monocytes (D, F, and H). Monocyte–macrophage differentiation of human cells was assessed by cell adhesion (C and E), cell size (C and E), and expression of macrophage marker gene, EMR1 (G). Monocyte–macrophage differentiation of mouse cells was assessed by cell adhesion (D and F), cell size (D and F), and expression of macrophage marker gene, F4/80 (H). *n* = 6 to 9; *P < 0.05 vs. control group.

As the expression of Morrbid was significantly increased during monocyte–macrophage differentiation, we thus created the lentivirus vectors expressing its shRNA (Lenti-Morrbid-Si) to test the role of Morrbid in monocyte–macrophage differentiation induced by differentiation stimuli: PMA for human THP-1 monocytes and M-CSF for mouse bone marrow-derived monocytes. As shown in Fig. 3A and B, Lenti-Morrbid-Si was successfully transfected into the cells and the expression of Morrbid in these human and mouse monocytes was significantly inhibited by Lenti-Morrbid-Si [20 multiple of infection (MOI)]. The effective transfection of Lenti-Morrbid-Si was also confirmed by fluorescence image of lentivirus-expressed Green Fluorescent Protein (GFP) in monocytes (Fig. S4). Interestingly, the differentiation stimuli-induced human THP-1 monocyte–macrophage differentiation (Fig. 3C, E, and G) and mouse monocyte–macrophage differentiation (Fig. 3D, F, and H) were significantly inhibited by Morrbid knockdown as shown by changes in (a) cell adhesion, (b) cell size, and (c) expression of macrophage marker genes EMR1 for human cells and F4/80 for mouse cells. It should be noted that we did not apply immunofluorescence to stain macrophages, which might provide more detailed information about the status of cell differentiation. The results suggested that Morrbid is not only a novel biomarker, but also a critical regulator for monocyte–macrophage differentiation.

**Fig. 4.**
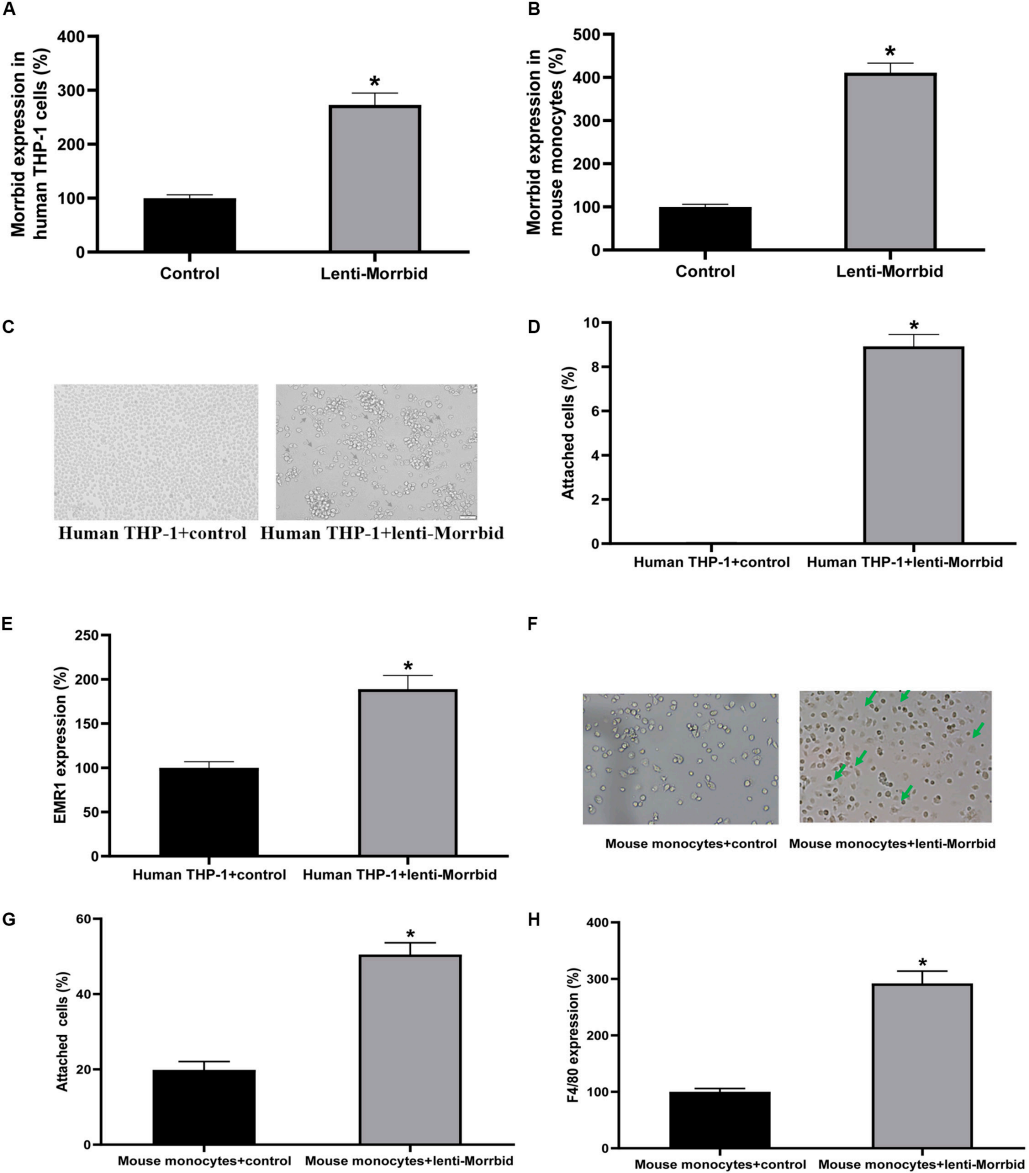
Morrbid alone is sufficient to elicit the monocyte–macrophage differentiation. (A) The expression of Morrbid in THP-1 cells was significantly increased by Lenti-Morrbid (20 MOI). (B) The expression of Morrbid in mouse bone marrow-derived monocytes was significantly increased by Lenti-Morrbid (20 MOI). Up-regulation of Morrbid via Lenti-Morrbid (20 MOI, 6 days) could induce macrophage differentiation of THP-1 cells as shown by increased adherence (C and D) and the increased differentiation marker gene EMR1 (E). Up-regulation of Morrbid via Lenti-Morrbid (20 MOI, 6 days) could induce macrophage differentiation of mouse monocytes as shown by increased cell size and adherence (F and G), as well as the increased differentiation marker gene F4/80 (H). Note: *n* = 6 to 9; *P < 0.05 compared with the control group.

To test whether Morrbid itself is sufficient to elicit the differentiation changes of monocytes, gain-of-function approach was applied in both human THP-1 monocytes and mouse bone marrow-derived monocytes without any differentiation stimuli. Morrbid in these monocytes could be successfully up-regulated by lentivirus vector expressing Morrbid (Lenti-Morrbid, 20 MOI) as shown in Fig. 4A and B. The cells were observed for up to 6 days after treatment with control lentivirus (Control, 20 MOI or Lenti-Morrbid, 20 MOI). Again, the monocyte–macrophage differentiation was evaluated by (a) cell adhesion, (b) cell size, and (c) expression of macrophage marker genes. The results demonstrated that Morrbid alone was sufficient to elicit the changes of monocyte–macrophage differentiation. Indeed, overexpression of Morrbid was able to induce the monocyte–macrophage differentiation both in human THP-1 monocytes (Fig. 4C to E) and in mouse monocytes (Fig. 4F to H). There are almost no attached human THP-1 monocytes in the control lentivirus-treated group; however, a significant number of THP-1 monocytes (about 10%) were attached after overexpression of Morrbid via Lenti-Morrbid without any differentiation stimuli (Fig. 4C and D). In addition, the cell size was increased (Fig. 4C), accompanied by the increased expression of macrophage marker gene (Fig. 4E). In bone marrow-derived mouse primary monocytes, the pro-differentiation effect of Morrbid was more pronounced, compared with that in THP-1 cells. Up to 6 days of Morrbid overexpression, the mouse monocyte size was significantly increased (Fig. 4F) and the attached cell reached as high as about 50% (Fig. 4G). Again, the expression of mouse macrophage marker gene F4/80 was increased after treatment with Lenti-Morrbid (Fig. 4H). Thus, the Morrbid-mediated effect on the monocyte–macrophage differentiation is not limited to stimuli-induced phenotypic change, and Morrbid might be a causative regulator of the monocyte–macrophage differentiation.

### Morrbid-deficient monocytes isolated from Morrbid knockout mice are resistant to the monocyte–macrophage differentiation

**Fig. 5.**
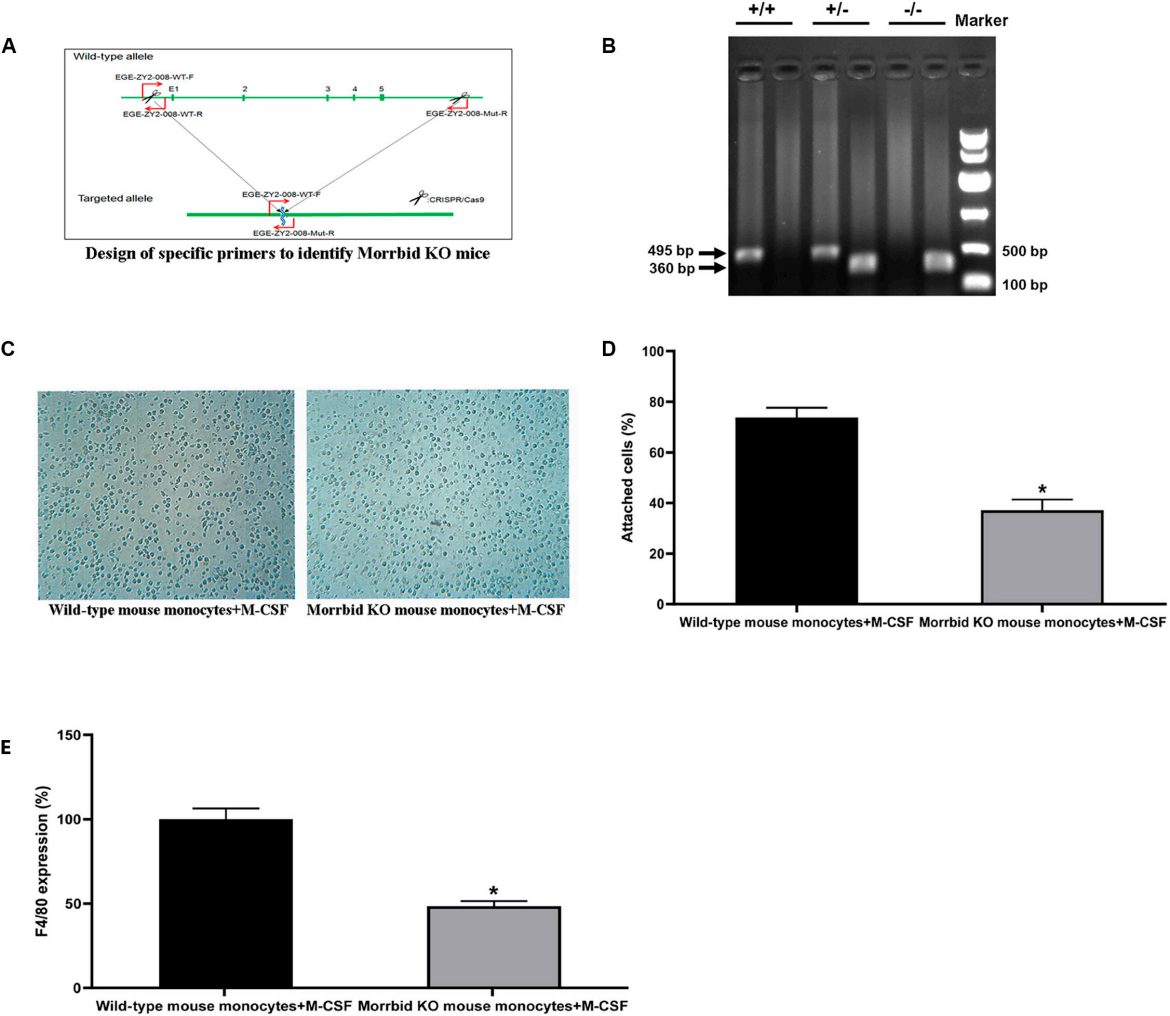
Morrbid-deficient monocytes from Morrbid knockout mice are resistant to the monocyte–macrophage differentiation in vitro. (A) The primer design to identified wild-type mice (Morrbid+/+), heterozygous Morrbid knockout mice (Morrbid−/+), and homozygous Morrbid knockout mice (Morrbid−/−). (B) Representative PCR bands from Morrbid+/+ mice (with a 459-bp band); Morrbid−/− mice (with a 360-bp band); and Morrbid−/+ mice (with both bands). The monocyte–macrophage differentiation induced by M-CSF (20 ng/ml, for 48 h) was significantly inhibited in Morrbid-deficient monocytes from the Morrbid knockout mice as shown by the decreased cell adherence (C and D) and the increased differentiation marker gene F4/80 (E). Note: *n* = 6; *P < 0.05 compared with the control group.

To further verify the role of Morrbid in monocyte–macrophage differentiation, we produced the Morrbid knockout mice through Biocytogen Co. Ltd. The successful knockout of Morrbid was verified via PCR by using specifically designed primers. The primers designed to identify wild-type mice (Morrbid+/+), heterozygous Morrbid knockout mice (Morrbid−/+), and homozygous Morrbid knockout mice (Morrbid−/−) are shown in Fig. 5A. The sequences of these primers are listed in Table S1. Using this method, Morrbid+/+ mice should have a 459-bp band, Morrbid−/− mice should have a 360-bp band, while Morrbid−/+ mice should have the above 2 bands. As shown in Fig. 5B, Morrbid was successfully knocked out as no Morrbid (459-bp band) was found in monocytes from the Morrbid knockout mice. Interestingly, the monocyte–macrophage differentiation induced by M-CSF (20 ng/ml, for 48 h) was significantly inhibited in these Morrbid-deficient monocytes from Morrbid knockout mice (Fig. 5C to E).

### Morrbid is a critical gene in monocyte–macrophage differentiation in mice

**Fig. 6.**
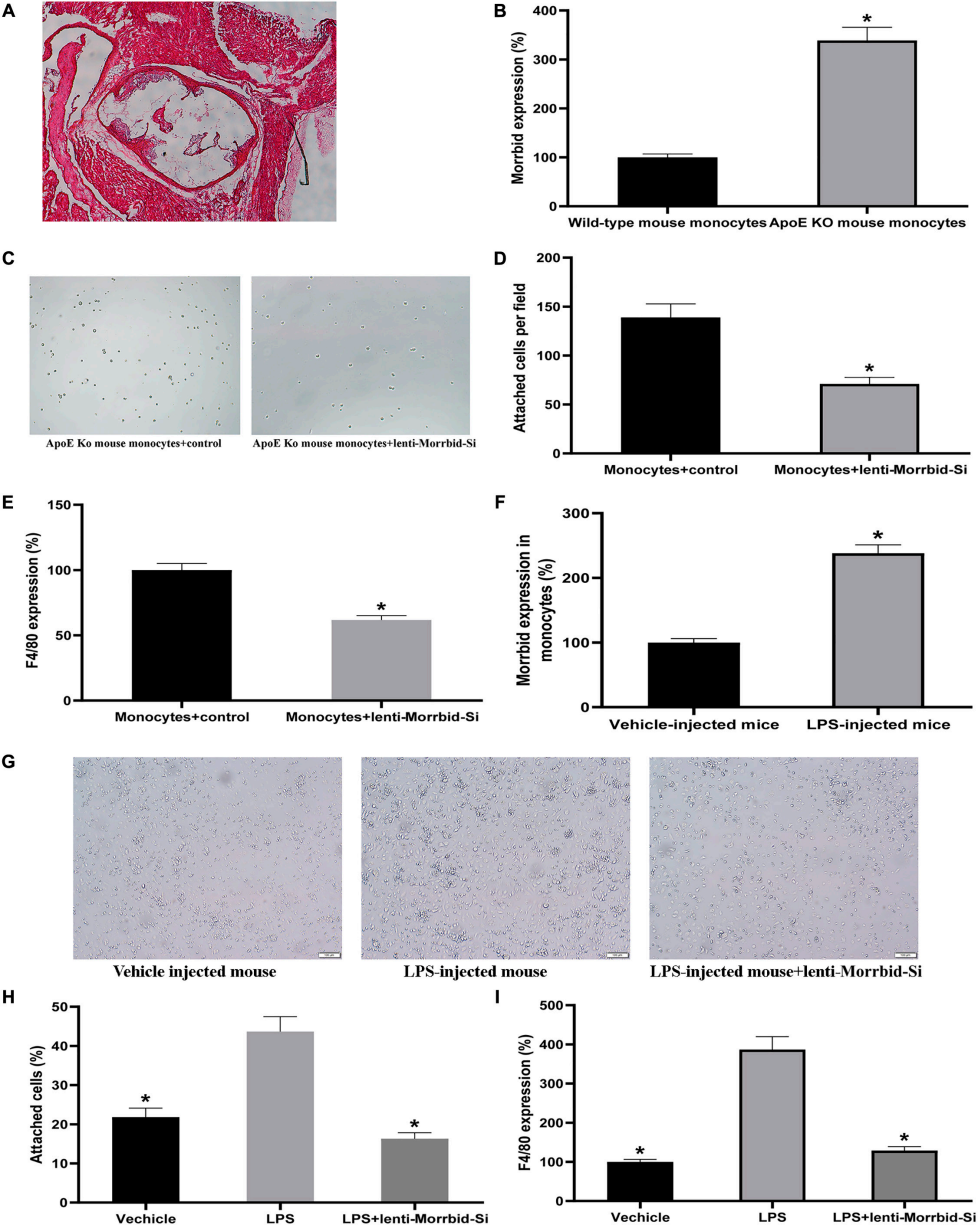
Morrbid is a critical gene in monocyte–macrophage differentiation in atherosclerotic mice and in mice with endotoxemia in vivo. (A) Representative H-E-stained section showing the atherosclerotic lesions in aortic root of ApoE knockout mice with the Western diet. (B) The expression of Morrbid in bone marrow-derived monocytes from ApoE mice with atherosclerosis was significantly increased. (C–E) The monocyte–macrophage differentiation of monocytes from atherosclerotic mice was inhibited by knockdown of Morrbid via Lenti-Morrbid-Si (20 MOI) as shown by the decreased adherence (C and D) and differentiation marker gene F4/80 (E). (F) The expression of Morrbid in bone marrow-derived monocytes from septic mice was increased. (G–I) The monocyte–macrophage differentiation was enhanced in these septic mice, which could be inhibited by knockdown of Morrbid via Lenti-Morrbid-Si (20 MOI) as shown by changes in cell adherence (G and H) and differentiation marker gene F4/80 (I). Note: *n* = 6 to 9; *P < 0.05 compared with the control group or LPS group.

It is well known that monocytes from atherosclerotic mice have the enhanced monocyte–macrophage differentiation. To provide an in vivo evidence regarding the role of Morrbid in monocyte–macrophage differentiation, we first applied ApoE knockout mice with atherosclerosis induced by a Western diet for 12 weeks as described in our previous publications [14,17]. As shown in Fig. 6A, atherosclerosis was induced in these ApoE knockout mice with the Western diet. Then, bone marrow-derived monocytes were isolated from these atherosclerotic ApoE knockout mice. Not surprisingly, consistent with the Morrbid expression in monocytes from CAD patients, the expression of Morrbid was increased in bone marrow-derived monocytes from ApoE mice with atherosclerosis compared with that in monocytes from wild-type normal control mice (Fig. 6B). Interestingly, the monocyte–macrophage differentiation of monocytes from atherosclerotic mice could be effectively inhibited by knockdown of Morrbid via Lenti-Morrbid-Si (20 MOI) as shown by the decreased adherence (Fig. 6C and D) and differentiation marker gene F4/80 (Fig. 6E). The result from ApoE knockout mice in vivo is consistent with that in cultured monocytes in vitro.

We also measured the expression levels of Morrbid in monocytes from LPS-induced endotoxemic mice. Consistent with the Morrbid expression in monocytes from patients with sepsis, the expression of Morrbid in bone marrow-derived monocytes from septic mice was increased compared with that in monocytes from mice without LPS injection (Fig. 6E). In addition, the monocyte–macrophage differentiation was enhanced in these septic mice, which could be inhibited by knockdown of Morrbid via Lenti-Morrbid-Si (20 MOI) (Fig. 6G–I).

### PI3-kinase/Akt is a signaling pathway regulating Morrbid expression in monocytes, while s100a10 is a downstream target gene of Morrbid that is involved in Morrbid-mediated effect on monocyte–macrophage differentiation

For the molecular mechanisms of Morrbid in monocytes, we wish to answer 2 questions: (a) Why was the expression of Morrbid increased in monocytes during monocyte–macrophage differentiation? (2) What was the downstream gene target of Morrbid that was responsible for Morrbid-mediated effect on monocyte–macrophage differentiation?

**Fig. 7.**
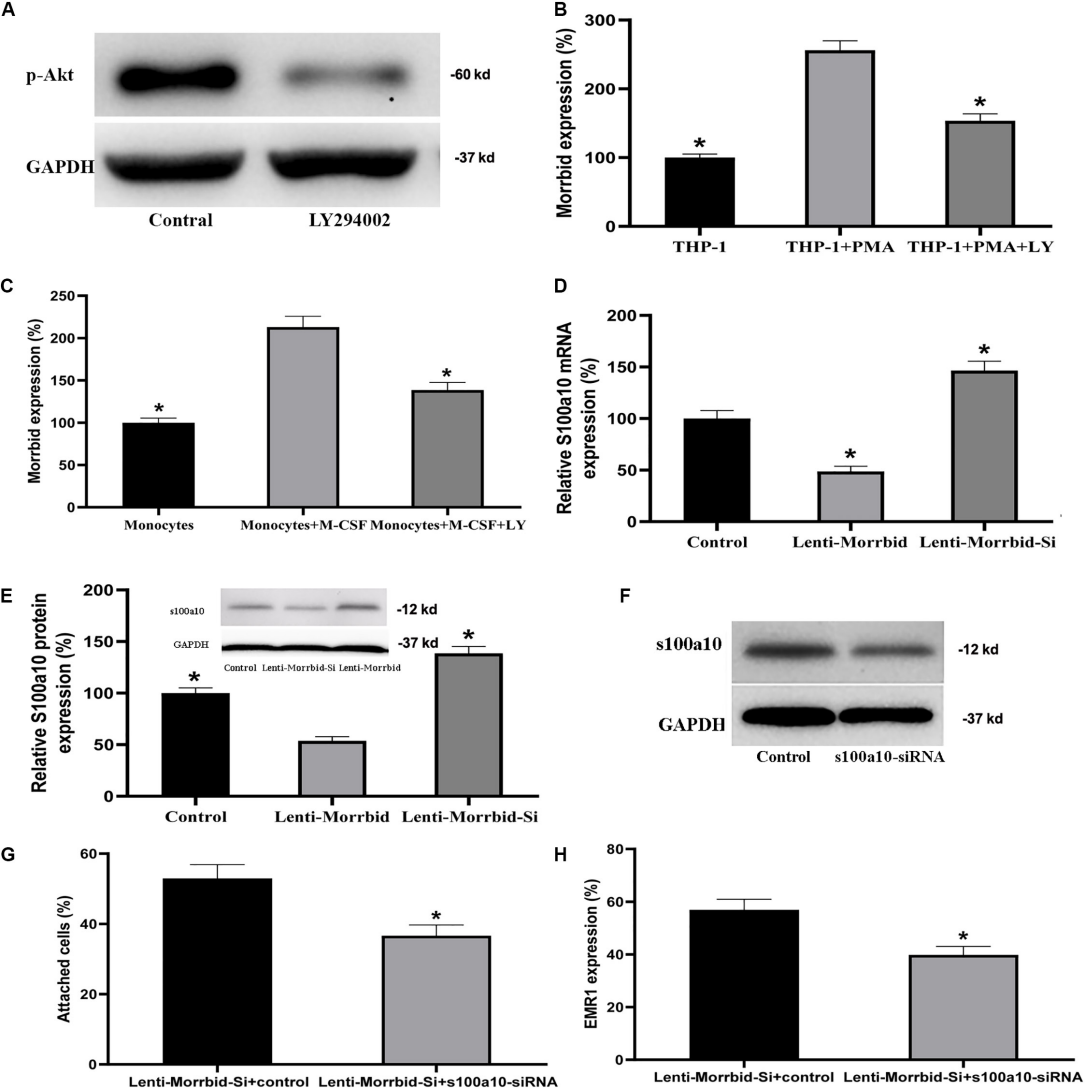
Upstream and downstream molecular mechanisms involved in Morrbid-induced effect on monocyte–macrophage differentiation. (A) The inhibitory effect of LY294002 on PI3-kinase/Akt was confirmed by Western blot, in which the phospho-Akt (p-Akt) was effectively inhibited. (B) The enhanced expression of Morrbid during macrophage differentiation of human THP-1 monocytes was inhibited by LY294002. (C) The enhanced expression of Morrbid during macrophage differentiation of mouse monocytes was inhibited by LY294002. (D) The expression of s100a10 in THP-1 cells was increased by Lenti-Morrbid-Si (20 MOI), but decreased by Lenti-Morrbid (20 MOI) at mRNA level. (E) The expression of s100a10 in THP-1 cells was increased by Lenti-Morrbid-Si (20 MOI), but decreased by Lenti-Morrbid (20 MOI) at the protein level. (F) Representative Western blots of s100a10 in THP-1 cells treated with siRNA control (control) or siRNA for s100a10 (s100a10-siRNA). (G) After s100a10 knockdown via s100a10-siRNA (100 nM), Lenti-Morrbid (20 MOI)-induced monocyte attachment was attenuated. (H) After s100a10 knockdown via s100a10-siRNA (100 nM), Lenti-Morrbid (20 MOI)-induced EMR1 expression in monocytes was attenuated. Note: *n* = 6 to 9; *P < 0.05 compared with the control group.

PI3-kinase/Akt is a well-known signaling pathway in monocyte–macrophage differentiation [19]. To test the potential regulatory role of PI3-kinase/Akt in the up-regulation of Morrbid during monocyte–macrophage differentiation, cultured human THP-1 monocytes and mouse monocytes were treated with vehicle or PI3-kinase/Akt pathway inhibitor LY294002 (20 μmol/L) [20]. Then, monocyte–macrophage differentiation was induced by PMA, and the expression of Morrbid was determined. The inhibitory effect of LY294002 on PI3-kinase/Akt was confirmed by Western blot, in which the phospho-Akt (p-Akt) was effectively inhibited (Fig. 7A). As shown in Fig. 7B and C, the increased expression of Morrbid during monocyte–macrophage differentiation was attenuated via LY294002. The results suggested that the PI3-kinase-Akt pathway was involved in the up-regulation of Morrbid expression during monocyte–macrophage differentiation.

To explore the target gene of Morrbid-mediating monocyte–macrophage differentiation, we used 1743 microarray data by Affymetrix GeneChip Human Genome U133 Plus 2.0 and annotated probe files linked to Morrbid and then analyzed by Multi Experiment Matrix (https://biit.cs.ut.ee/mem/index.cgi). We selected 100 genes that were closely related with Morrbid expression in different tissues or cells from both human and mice. Based on the frequency of these genes, we select 7 candidate genes to find the relationship with Morrbid expression. We considered that the Morrbid target genes should comply with the following 2 requirements: (a) it is related to the differentiation of monocyte–macrophage, and (b) its expression could be regulated by Morrbid. Among the 7 candidate genes, bioinformatics analysis revealed that s100a10 mRNA had the Morrbid binding site. We thus tested the s100a10 expression in THP-1 cells with Morrbid expression modulation (overexpression via Lenti-Morrbid or knockdown via Lenti-Morrbid-Si). As shown in Fig. 7D and E, both s100a10 mRNA and protein were up-regulated by Morrbid overexpression but were down-regulated by Morrbid knockdown. In addition, to test the role of s100a10 in Morrbid-mediated monocyte–macrophage differentiation, s100a10 expression was successfully inhibited by s100a10 siRNA transduction (Fig. 7F). In the s100a10-down-regulated monocyte, the attached ratio of cells and EMR1 expression induced by Lenti-Morrbid (20 MOI) was significantly attenuated (Fig. 7G and H). In addition, s100a10 knockdown via s100a10 siRNA could also inhibit macrophage differentiation of THP-1 monocytes induced by PMA (Fig. S5). The results suggest that s100a10 is a downstream target gene that is responsible for the Morrbid-mediated effect on monocyte–macrophage differentiation.

### Overexpression of Morrbid enhances but monocyte/macrophage-specific knockout of Morrbid inhibits atherosclerotic lesion formation and the recruitment of monocytes and macrophages in mouse carotid arteries of the acute atherosclerosis model

The enhanced monocyte–macrophage differentiation is related to increased infiltration and recruitment of monocytes and macrophages into arterial walls and the followed atherogenesis. To test the effects of Morrbid on the recruitment of monocytes and macrophages and atherosclerosis in vivo, acute atherosclerosis was induced by partial left carotid artery ligation (PLCA) and the Western diet for 3 weeks in ApoE knockout mice [21,22]. In this model, 3 of 4 caudal left carotid artery branches (left external carotid, internal carotid, and occipital arteries) were ligated. We have confirmed that this acute atherosclerosis model has the increased Morrbid levels in the atherosclerosis carotid arteries as shown in Fig. S6.

**Fig. 8.**
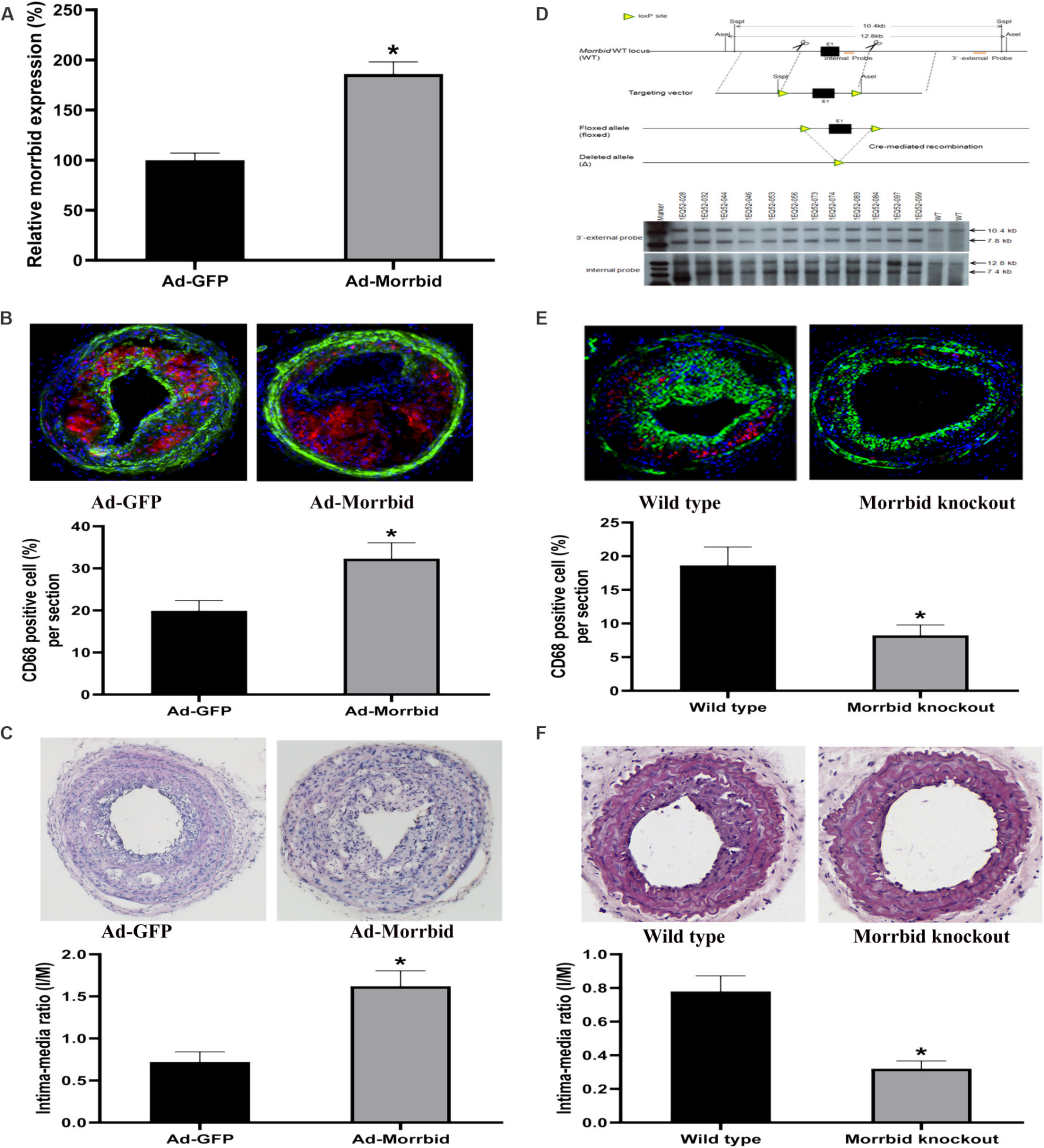
Overexpression of Morrbid increases but leukocyte-specific Morrbid knockout inhibits atherosclerotic lesion formation and the recruitment of monocytes and macrophages in mouse carotid arteries of the acute atherosclerosis model. (A) Ad-Morrbid (5 × 10^9^ pfu/mouse) injection increased the expression of injured mouse carotid arteries compared with that from the Ad-GFP group (control). (B and C) Overexpression of Morrbid enhanced the numbers of the recruited monocytes and macrophages (red color) and atherosclerotic lesions in injured vessels. (D) Leukocyte-specific Morrbid knockout mouse. (E and F) Leukocyte-specific Morrbid knockout inhibited the numbers of the recruited monocytes and macrophages (red color) and atherosclerotic lesions in injured vessels. *P < 0.05 compared with the control group.

To increase the expression of Morrbid in vivo, Ad-Morrbid (5 × 10^9^ pfu/mouse) or its control Ad-GFP (5 × 10^9^ pfu/mouse) was injected into ApoE^−/−^ mice via external jugular vein. The successful up-regulation of Morrbid in injured carotid arteries at 1 week (for Ad-Morrbid) after PLCA was confirmed by quantitative real time polymerase chain reaction (qRT-PCR) (Fig. 8A). Interestingly, the numbers of the recruited monocytes and macrophages (CD68 positive cells) in injured vessels from Morrbid overexpressed (Ad-Morrbid) mice were significantly higher than those from the control animals (Fig. 8B). In addition, overexpression of Morrbid increased atherosclerotic lesion formation at 3 weeks after PLCA and Western diet (Fig. 8C).

To further confirm the role of Morrbid in monocyte–macrophage differentiation and atherogenesis in vivo, a loss-of-function approach via monocyte/macrophage-specific Morrbid knockout mice was applied. The method to create the monocyte/macrophage-specific Morrbid knockout mice and the confirmation for the successful knockout of Morrbid in monocytes and macrophages are described in the Materials and Methods section and are shown in Fig. 8D. Clearly, the numbers of the recruited monocytes and macrophages in injured vessels from the monocyte/macrophage-specific mice were lower than those from the control wild-type animals (Fig. 8E). In addition, knockout of Morrbid in monocyte/macrophage inhibited atherosclerotic lesion formation in the acute atherosclerosis model (Fig. 8F).

## Discussion

It is well-known that monocyte–macrophage differentiation is a key cellular event in normal development, physiological homeostasis, tissue repair, and immune defense. In addition, under diverse disease conditions such as infection/inflammation, cardiovascular disease, metabolic disorders, and tumors, the balance of monocyte–macrophage differentiation is disrupted and the abnormal monocyte–macrophage differentiation plays important roles in these diseases [1–4,21,22]. Despite intensive clinical and laboratory studies on the monocyte–macrophage differentiation and its disruption, the molecular mechanisms involved remain largely unknown [5,6,22]. To date, there are no good methods to control the monocyte–macrophage differentiation due to our limited knowledge about its molecular mechanisms [5,6,23,24]. It is therefore very important to study the novel mechanisms and to identify new therapeutic molecular targets of the abnormal monocyte–macrophage differentiation [24].

Increasing studies have suggested lncRNAs play critical roles in normal development, and physiological and pathological processes, in which either normal or abnormal monocyte–macrophage differentiation is involved [10–12]. However, the biological roles of lncRNAs in monocyte–macrophage differentiation are still unclear. The long-time goal of our research program is to determine the biological roles of leukocyte-enriched lncRNAs in monocyte–macrophage differentiation and its related vascular diseases. To select the leukocyte-enriched lncRNAs that we studied, we applied the following selective parameters: (a) they should be among the 20 most abundant lncRNAs in human monocytes [25]; (b) they should be among the 10 lncRNAs with the highest expression changes during monocyte-to-M0 macrophage differentiation; and (c) they should be conserved between human and mouse. By crossover analysis of these 3 parameters, only 2 lncRNAs were identified, one was Morrbid, and another one was AK050834 (we named it as Rommd). We found that Morrbid expression was increased during monocyte-to-M0 macrophage differentiation. In contrast, the Rommd expression was significantly decreased during monocyte-to-M0 macrophage differentiation (Fig. S7).

In this study, we were focused on the study of Morrbid, which was a recently identified leukocyte-specific lncRNA [13]. The genetic locations, sequences, and the good conservation of human Morrbid and mouse Morrbid are demonstrated in Fig. S8. We determined, for the first time, the expression changes of Morrbid in monocytes from patients with atherosclerotic CAD, a well-known human disease with the enhanced monocyte–macrophage differentiation. We found that Morrbid expression in monocytes from CAD patients was higher than that from control healthy subjects. In addition, the expression of Morrbid in atherosclerotic mouse and human arterial walls was significantly increased compared with that in control vessels.

To test whether the aberrant expression of Morrbid in monocytes is CAD specific or is a general molecular phenomenon in human monocytes with the enhanced differentiation from monocyte to macrophage, we also determined the Morrbid expression levels in monocytes from patents with sepsis, which is another human disease with the enhanced monocyte–macrophage differentiation. We obtained a similar result with CAD patients. It is clear that the increased Morrbid expression could be a general epigenetic change in monocytes with the enhanced monocyte–macrophage differentiation. Thus, Morrbid might be a novel biomarker for the status of monocyte–macrophage differentiation, although additional studies need to be performed to confirm it under other disease conditions.

The above results from patients encouraged us to determine the role of Morrbid in monocyte–macrophage differentiation using the well-established cultured cell models. In human THP-1 monocytes, we found that Morrbid expression was significantly increased in these cells during monocyte–macrophage differentiation induced by PMA. The monocyte–macrophage differentiation was effectively inhibited by Morrbid inhibition. THP-1 cells are immortalized human monocytes; to avoid any unpredictable effects of immortalization, we performed similar experiments in human primary PBMCs. The results were consistent with those from THP-1 cells. To provide the association of Morrbid with monocyte–macrophage differentiation in other species, and to provide an evidence for our in vivo intervention studies, we also performed similar experiments in mouse primary monocytes and obtained similar results with human cells. Thus, Morrbid is a critical gene in the control of monocyte–macrophage differentiation in cultured human and mouse cells in vitro. More importantly, we found that overexpression of Morrbid alone was sufficient to elicit the monocyte–macrophage differentiation without any differentiation stimuli. Thus, Morrbid might be a causative regulator of the monocyte–macrophage differentiation. Finally, the critical role of Morrbid in monocyte–macrophage differentiation was verified by using the Morrbid-deficient monocytes from Morrbid knockout mice.

To verify the role of Morrbid in monocyte–macrophage differentiation in vivo, we first applied ApoE knockout mice with atherosclerosis. Interestingly, the enhanced monocyte–macrophage differentiation in atherosclerotic mice was able to be reversed by Morrbid knockdown. To provide additional proof of concept in vivo, we also applied the LPS-induced endotoxemic (septic) mice. In this infection model, we also found that monocytes from LPS-injected mice had the increased Morrbid expression with the enhanced monocyte–macrophage differentiation, which could also be effectively reversed via Morrbid knockdown. Thus, Morrbid is also a critical modulator for monocyte–macrophage differentiation in vivo.

It was clear that Morrbid expression was increased in monocytes during their differentiation into macrophages. The challenging question is why? To address this issue, we have performed the detailed analysis about the signaling pathways related to the monocyte–macrophage differentiation in current available database and found that PI3-kinase/Akt signaling pathway is one of the most important pathways in monocyte–macrophage differentiation. By using the signaling pathway inhibitor, we found that PI3-kinase-Akt was a signaling pathway in the regulation of Morrbid expression in monocytes. However, it was still unclear how the PI3-kinase/Akt achieved its effect on Morrbid expression, which needs to be studied in the future. Nevertheless, PI3-kinase/Akt/Morrbid may represent a novel signaling pathway in cells. In addition, inhibition of PI3-kinase/Akt could not completely block the abnormal up-regulation of Morrbid, suggesting that other signaling pathway(s) may also participate in the expression regulation of Morrbid in monocyte and macrophages.

It is known that an lncRNA has its biological functions via its multiple target genes. S100a10 is one of the S100 family members of calcium-binding proteins. Several recent studies have suggested that S100a10 is a critical gene for cancer cell migration, as well as the tumor-promoted macrophage migration [26–28]; however, its role in monocyte–macrophage differentiation has not been reported. In this study, we found that s100a10 was a target gene of Morrbid, which was involved, at least in part, in Morrbid-mediated biological effect on monocyte–macrophage differentiation. Indeed, the Morrbid overexpression-mediated biological effect on monocyte–macrophage differentiation was partially inhibited and affected via s100a10 blocking. Morrbid was able to affect the expression of S100a10 at both mRNA and protein levels. Our data suggested that the binding of Morrbid with s100a10 mRNA could be a major regulatory mechanism by which Morrbid could affect the expression of s100a10. Whether the Morrbid could bind with S100a10 protein to affect its expression should be tested in the future. In addition, other unknown target genes of Morrbid that are related to monocyte–macrophage differentiation should be determined in future studies. Nevertheless, s100a10 as a new target of Morrbid and the effect of s100a10 in monocyte–macrophage differentiation are new discoveries in Morrbid, s100a10, and leukocyte biological research fields. We did find that overexpression of Morrbid could enhance while Morrbid knockdown could reduce the monocyte survival (Fig. S9). However, cell apoptosis/survival was not our focus in this study.

Finally, to provide a direct proof of concept of Morrbid in pathogenesis of monocyte/macrophage differentiation-related disease in vivo, we applied the acute atherosclerosis model in mice induced by PLCA and the Western diet. The result revealed that overexpression of Morrbid enhanced atherosclerotic lesion formation and the recruitment of monocytes and macrophages in mouse carotid artery in this atherosclerosis model. In contrast, monocyte/macrophage-specific knockout of Morrbid inhibited atherosclerotic lesion formation and the recruitment of monocytes and macrophages in the mouse atherosclerosis model. Thus, Morrbid indeed could participate in atherogenesis via modulating leukocyte phenotype and function in vivo. It should be noted that although the expression level of Morrbid is the highest in monocytes/macrophages, other cells, such as vascular smooth muscle cells and endothelial cells, in atherosclerotic lesions also express low level of Morrbid. The biology role of Morrbid in these cells from atherosclerotic lesions should be determined in the future.

In summary, we identified for the first time that the expression of the monocyte-enriched lnRNA Morrbid is increased in monocytes with the enhanced monocyte–macrophage differentiation, which is related to the PI3-kinase-Akt signaling pathway. The increased Morrbid is a critical inducer for the monocyte–macrophage differentiation and monocyte–macrophage differentiation-related atherogenesis via its target gene(s) such as s100a10 (Fig. S10). Morrbid is thus a novel biomarker and a modulator of monocyte–macrophage phenotypes, which is involved in atherogenesis. The study thus provides a novel molecular mechanism in macrophage differentiation, a new molecular link between leukocytes and inflammatory vascular diseases and a novel therapeutic target for atherosclerosis.

## Materials and Methods

### Mice

All mice were bred and maintained at the Southwest Medical University. Mice (between 5 and 12 weeks of age) were used for all experiments. C57BL/6 (wild type) and ApoE knockout mice (C57BL/6 background) were from The Jackson Laboratory. All the animal surgery and animal model were induced under general anesthesia with vaporized isoflurane (2%). At the end of all the animal experiments, the animals were euthanized with an overdose of sodium pentobarbital (200 to 250 mg/kg IP). The procedures of anesthesia and euthanizing method were consistent with the *Guide for the Care and Use of Laboratory Animals* (updated (2011) version of the NIH guidelines).

### Generation of global Morrbid knockout mice

We generated Morrbid-deficient mice by using the CRISPR/Cas9 system [13] with the help from Biocytogen Co. Ltd. We designed single guide RNAs (sgRNAs) against regions flanking the first and last exon of the Morrbid locus. The resulting founder mice by Cas9-mediated double-stranded DNA breaks were Morrbid−/+. The Morrbid−/+ mice were then intercrossed to obtain homozygous Morrbid−/− mice. Mice were crossed for at least 5 generations to wild-type mice and then intercrossed to obtain homozygosity. Littermate controls were used in our experiments.

### Generatizon of monocyte/macrophage-specific Morrbid knockout mice

To generate Morrbid^fl/fl^ mice on C57BL/6 background, we designed the Cas9/guide RNA (gRNA) target sequences and the 2 loxP sites are shown in Fig. 8D. Cas9 mRNA and sgRNAs were transcribed with T7 RNA polymerase in vitro. Cas9 mRNA, sgRNAs, and donor vector were mixed at different concentrations and co-injected into the cytoplasm of fertilized eggs at the one-cell stage. The Morrbid^fl/fl^ mice were validated by PCR amplification, direct sequencing, and Southern blot analysis with the probes indicated in Fig. 8D. By crossing Morrbid^fl/fl^ and LysM-Cre mice that express Cre-recombinase in the monocyte/macrophage lineage, we obtained monocyte/macrophage-specific Morrbid knockout (LysM-cre/Morrbid^fl/fl^) mice. In BM-derived monocytes and macrophages, we have confirmed that the expression was reduced to about 10% of WT control.

### Chronic mouse atherosclerosis model

Atherosclerosis was induced in the aortas of ApoE-knockout mice (C57BL/6 background) (The Jackson Laboratory) with a Western diet (21% fat, 0.15% cholesterol, and 19.5% casein) (TestDiet, Richmond, IN) for 12 weeks as described [18,19] (Fig. 6A). The wild-type C57BL/6 mice with the normal chow diet at the same age were used as the group. Atherosclerotic lesions were measured by oil red O staining of whole aortas and by hematoxylin-eosin (H-E) staining of the aortic root as shown in our previous publications [18,19]. The lesion areas were calculated by using an image software (Image-Pro Plus 6.0, Media Cybernetics, Rockville, MD).

### Acute mouse atherosclerosis model

Acute atherosclerosis was induced by PLCA and a Western diet (17.5% protein, 20% fat, and 0.15% cholesterol, TestDiet) for 3 weeks in ApoE knockout mice (C57BL/6 background, The Jackson Laboratory) as described [22,23]. In this model, 3 of 4 caudal left carotid artery branches (left external carotid, internal carotid, and occipital arteries) were ligated.

### Human subjects

This human study was approved by the institutional review committee of Southwest Medical University and Guangdong General Hospital. All the participants were given the informed consent, which is conformed to the Declaration of Helsinki. A total of 30 patients with atherosclerotic CAD were recruited from the Guangdong General Hospital. All diagnoses of CAD patients were confirmed with angiography. A total of 30 age- and sex-matched healthy controls were from the Health Examination Center and had no history of CAD and cerebrovascular diseases. Human atherosclerotic arteries were acquired from 6 arteriosclerosis obliterans patients, and the 6 normal artery samples were from donors without arteriosclerosis obliterans [18].

### Isolation of human PBMCs

Human PBMCs were isolated by density centrifugation and cultured in RPMI 1640 medium. The PBMCs were verified by fluorescence-associated cell sorting makers (CD14+CD3−CD16−CD56−).

### Isolation of mouse monocytes from bone marrow

The mouse bone marrow monocytes were isolated from mouse bone marrows as described [16,17]. In brief, monocytes were isolated from bone marrow of mouse femur and tibia. By removing adhesive macrophages, the purified monocytes were obtained and cultured for this study. The isolated monocytes were verified by fluorescence-activated cell sorting.

### Cell models of monocyte–macrophage differentiation

Three different monocytes were applied to induce monocyte–macrophage differentiation: human immortalized THP-1 cells, human primary monocytes (PBMCs), and mouse primary monocytes. THP-1 cells were differentiated into M0 macrophages by 48- to 72-h incubation with PMA (100 ng/ml). The differentiation of monocyte to macrophage was determined by morphological changes, the enhanced adherence (attached cells), and the increased expression of EMR1 and/or CD36. For the PBMCs, the macrophage differentiation was induced with M-CSF (50 ng/ml) for 48 to 72 h and was determined by morphological changes, the enhanced adherence, and the increased expression of CD71 and CD68 [4,15]. For the mouse primary monocytes, the macrophage differentiation was induced by M-CSF (20 ng/ml) for 48 to 72 h and was measured by morphological changes, the enhanced adherence, and the increased expression of F4/80 [15].

### Gene modulation in cultured cells

(a) Generation of lentivirus vector expressing Morrbid. Morrbid was constructed into the lentivirus expression vector (Lenti-Morrbid) by using a lentivirus expressing system (Applied Biological Materials Inc.). The lentiviral containing a control scramble sequence was used as control virus (Lenti-control). All the recombinant lentiviral vectors carried a GFP. (b) Generation of lentivirus vector expressing Morrbid shRNA sequence. Morrbid shRNA sequence was constructed into the lentivirus expression vector to generate lentivirus expressing Morrbid shRNA (Lenti-Morrbid-si). The lentivirus containing a control scramble sequence (OriGene) was used as control virus (Lenti-si-control). All the recombinant lentivirus vectors carried a GFP. The viral particles were produced by third-generation packaging in 293FT cells. (c) siRNA for s100a10 (S100a10 siRNA) and their controls were purchased from Life Technologies. S100a10 siRNA (S100a10 siRNA) and their controls were transfected into the cells by using a transfection reagent, TransIT X2. For the lentivirus vectors, the cells in the culture wells were transfected with these vectors at 20 MOI.

### Gene modulation in mice

To modulate the Morrbid expression in mice in vivo, Adenovirus vector expressing Morrbid (Ad-Morrbid) (5 × 10^9^ pfu/mouse) (Applied Biological Materials Inc.), Ad-GFP (control) (5 × 10^9^ pfu/mouse) (Applied Biological Materials Inc.), siRNA control (7 nmol) (Dharmacon, Inc.), or Morrbid-siRNA (7 nmol) (Dharmacon, Inc.) were injected into ApoE^−/−^ mice via external jugular vein. The siRNA control or Morrbid-siRNA was delivered via the in vivo jetPEI (Polyplus-transfection, France) at a ratio of 6 (total volume of the complex was 200 μl). The sequences for the Morrbid-siRNA were as follows: Sense, 5′ GCGCAUAUCUUGGGAUCUUUU 3′; Antisense, 5′ AAGAUCCCAAGAUAUGCGCUU 3′.

### RNA isolation and qRT-PCR

RNAs were isolated with an RNA Isolation Kit. qRT-PCR was performed on cDNA generated from 200 ng of total RNA using the protocol of a qRT-PCR mRNA Detection Kit (Roche). GADPH was used for template normalizations. Fluorescent signals were normalized to an internal reference, and the threshold cycle (Ct) was set within the exponential phase of the PCR. The target PCR Ct values were normalized by subtracting the GADPH Ct value, which provided the ΔCt value. The relative expression level between treatments was then calculated using the following equation: relative gene expression = 2^−(ΔCtsample−ΔCtcontrol)^ [11]. All the PCR primers are listed in Table S1.

### Western blot analysis

Western blot analysis was performed by using antibodies for p-Akt (1:2,000 dilution; Cell Signaling Technology, Danvers, MA) and s100a10 (1:2,000 dilution; Cell Signaling, Danvers, MA). As a loading control, GAPDH antibody (1:2,000 dilution; Cell Signaling, Danvers, MA) was used.

### Statistical analysis

All data will be presented as mean ± standard error. All the experiments were repeated independently at least 3 times. For relative gene expression, the mean value of control group is defined as 100% or 1. SPSS was used to perform the statistical analysis. One-way ANOVA within groups was used to assess the significance of any change between groups. Comparisons between 2 groups were performed using the independent samples *t* test. The results were considered to be statistically significant when *P* < 0.05.

### Ethics statement

The human study was approved by the institutional review committee of Southwest Medical University and Guangdong General Hospital, and the participants were given informed consent, which conformed to the Declaration of Helsinki. All animal studies were approved by the animal care committee at Southwest Medical University.

## Data Availability

The data are available from the authors upon a reasonable request.

## Supplementary Materials

Supplementary 1 Supplementary material is available at the online version.
