# Supplementary material for: Leukocyte-Specific Morrbid Promotes Leukocyte Differentiation and Atherogenesis
Source: Research (Wash D C). 2023 Jul 6;6:0187. doi: 10.34133/research.0187 (PMC10325668; doi:10.34133/research.0187)
Supplement: Supplementary 1 — Supplementary material is available at the online version. [file research.0187.f1.pdf]

Supplemental Data

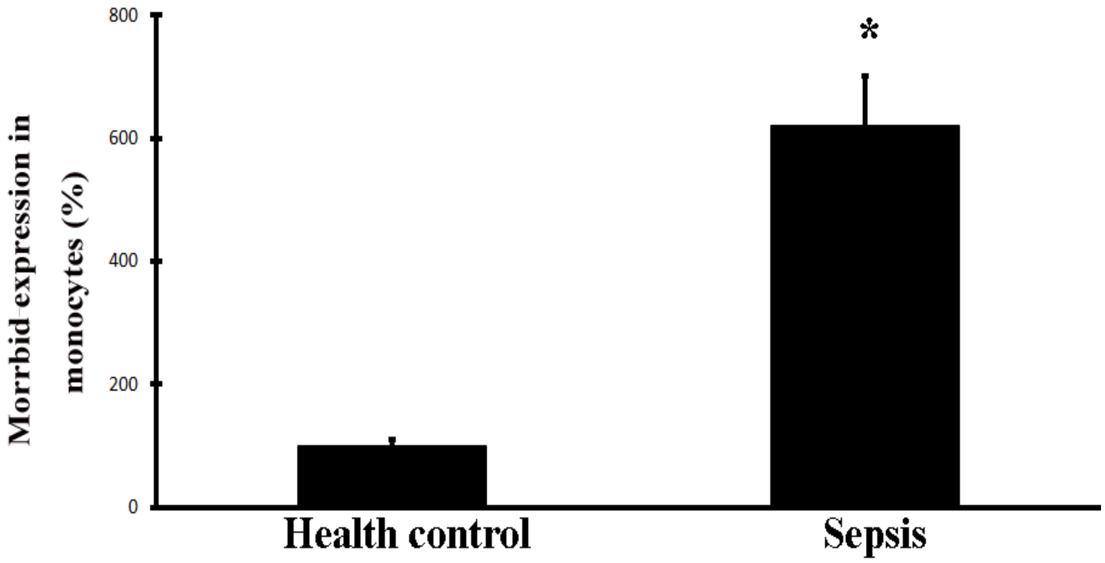

Supplemental Figure 1. *Morrbid* expression in monocytes is increased from patients with sepsis. Note: n=12; *p<0.001 compared with the control group.

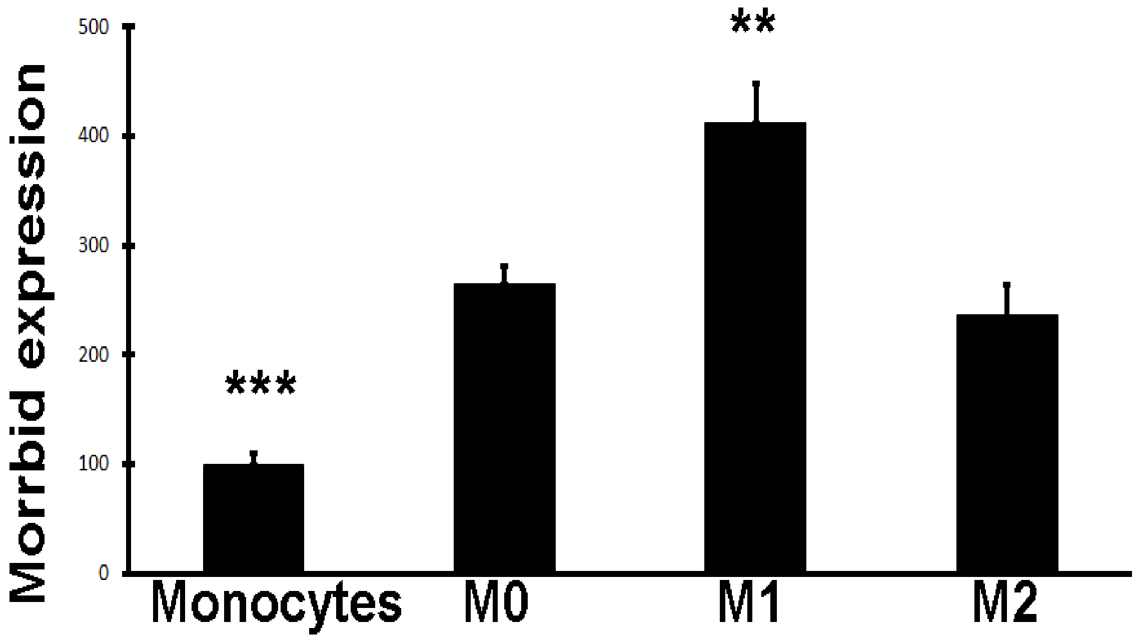

Supplemental Figure 2. Relative *Morrbid* expression in monocytes, M0 macrophages, M1 macrophages, and M2 macrophages. n=6; **p<0.01, ***p<0.001 vs. M0 group.

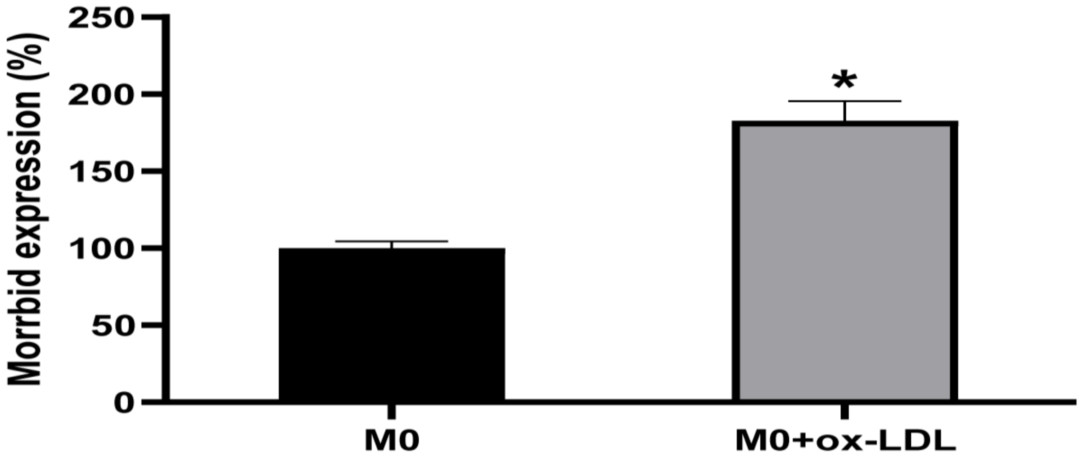

Supplemental Figure 3. Relative *Morrbid* expression in M0 macrophages and in M0 macrophages after treatment with the oxidized low-density lipoprotein (oxLDL) (80μg/ml). n=6; *p<0.01 vs. M0 group.

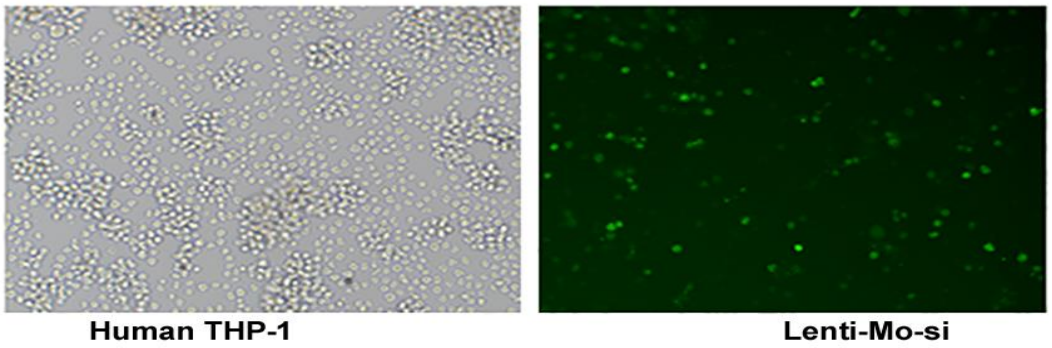

Supplemental Figure 4. Effective transfection of Lenti-Morrbid-Si in human THP-1 cells. Human THP-1 cells were treated with Lenti-Morrbid-Si expressing GFP (20 MOI). Left: Human THP-1 cells; Right: Lenti-Morrbid-Si (Lenti-Mo-si) (green).

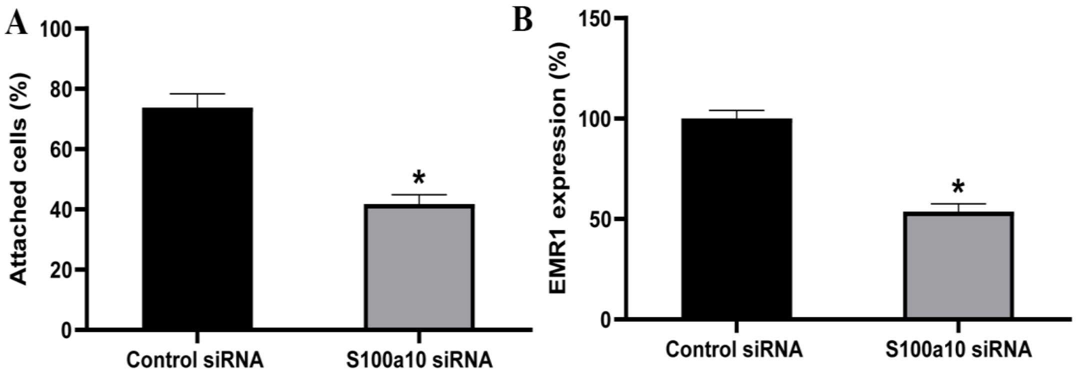

Supplemental Figure 5. The effect of s100a10 knockdown on the macrophage differentiation of THP-1 monocytes. s100a10 knockdown via s100a10 siRNA could inhibit macrophage differentiation of THP-1 monocytes induced by PMA as shown by the decreased cell attachment (A) and EMR1 expression (B). n=6; *p<0.01 vs. M0 group.

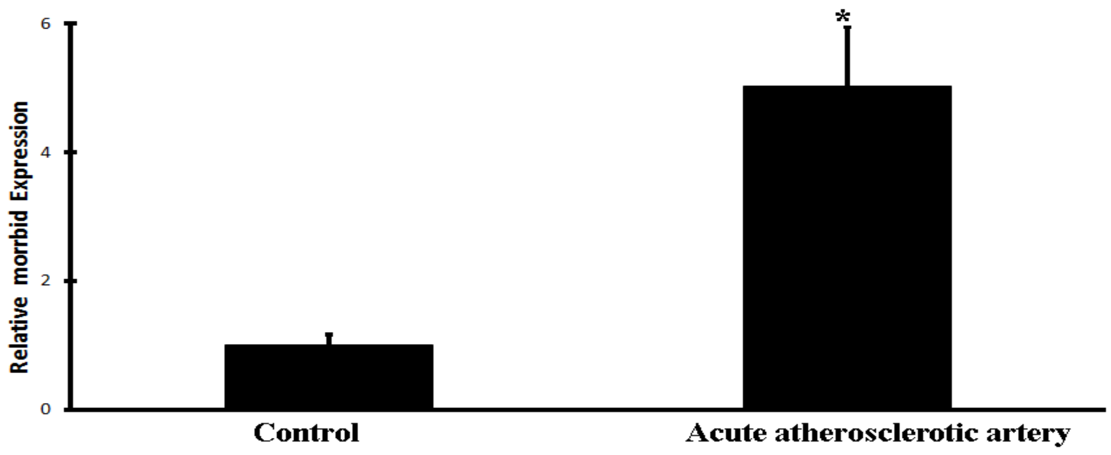

Supplemental Figure 6. The expression of in mouse carotid arteries is increased in the acute atherosclerotic model. Note: n=12, p<0.001.

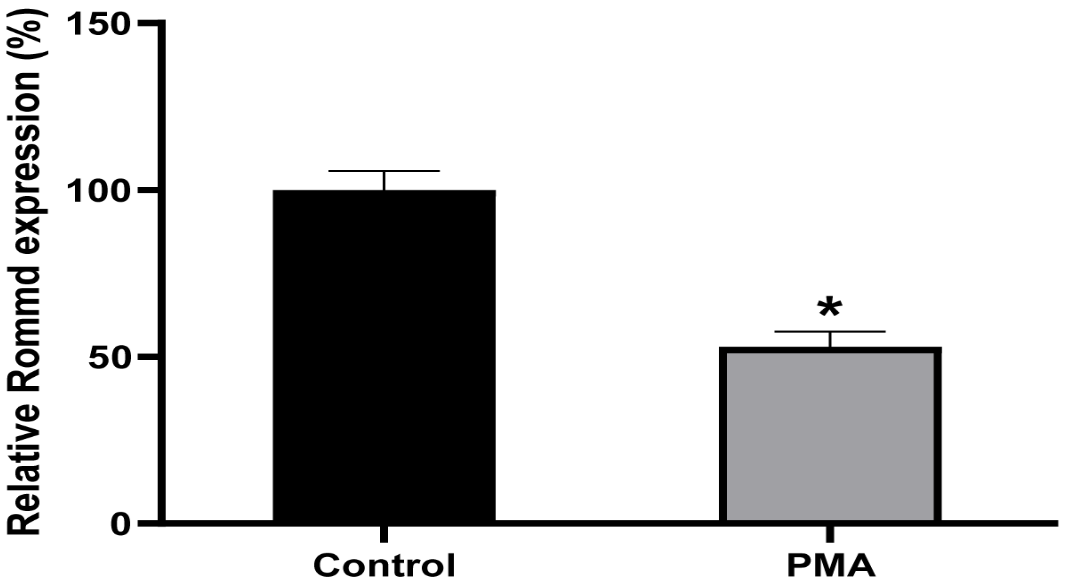

Supplemental Figure 7. Expression change of Rommd during monocyte to M0 macrophage differentiation. The Rommd expression was decreased during monocyte to M0 macrophage differentiation. n=6; *p<0.01 vs. M0 group.

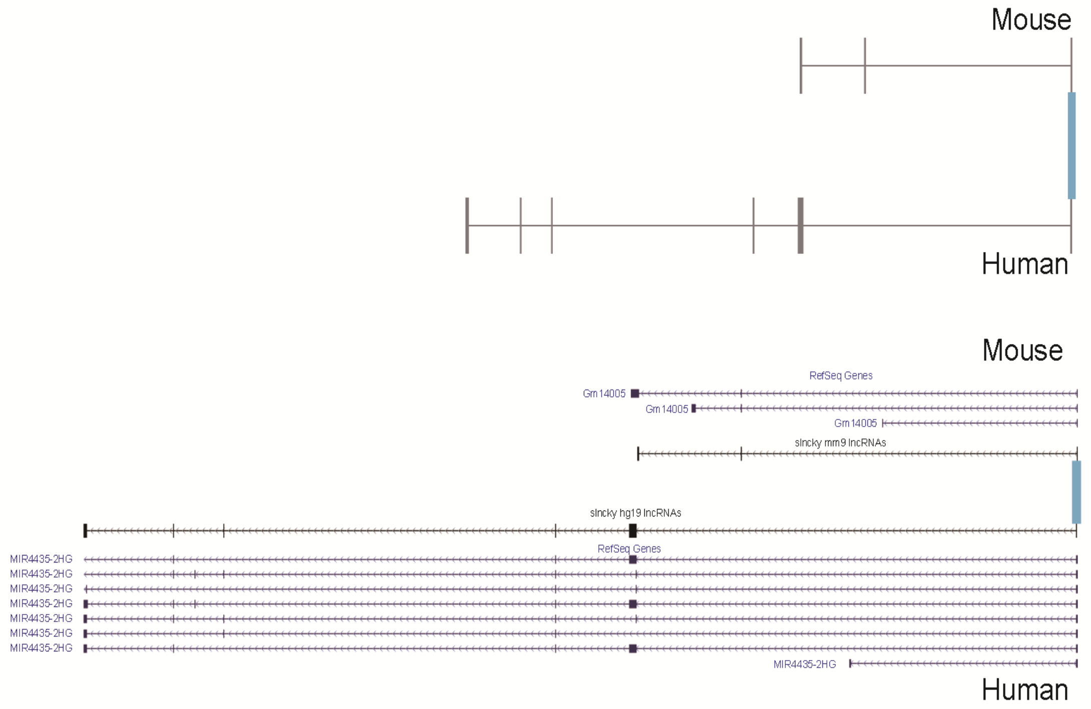

1). Gene Information about the Morrbid in mouse (Gm14005)
Gene type: lncRNA
Organism: Mus musculus
Location: 2:128,178,319-128,502,765
Exon count: 5

2). Gene Information about the Morrbid in human (MIR4435-2HG)
Gene type: lncRNA
Organism: Homo sapiens
Location: 2:111,196,350-111,495,100

3) Homologous analysis between Human and mouse Morrbid
is calculated by slncky software through website
https://scripts.mit.edu/~jjenny.
Slncky can characterize sequence and transcript conservation
properties of orthologous lncRNAs, which bases on four metrics:
(1) A ‘transcript-genome identity’(TGI) score,
(2) A ‘transcript-transcript identity’ (TTI) score,
(3) A ‘splice site conservation’ (SSC) score,
(4)An ‘insertion/deletion rate.

Supplemental Figure 8. The genetic locations, sequences, and the good conservation of human *Morrbid* and mouse *Morrbid.*

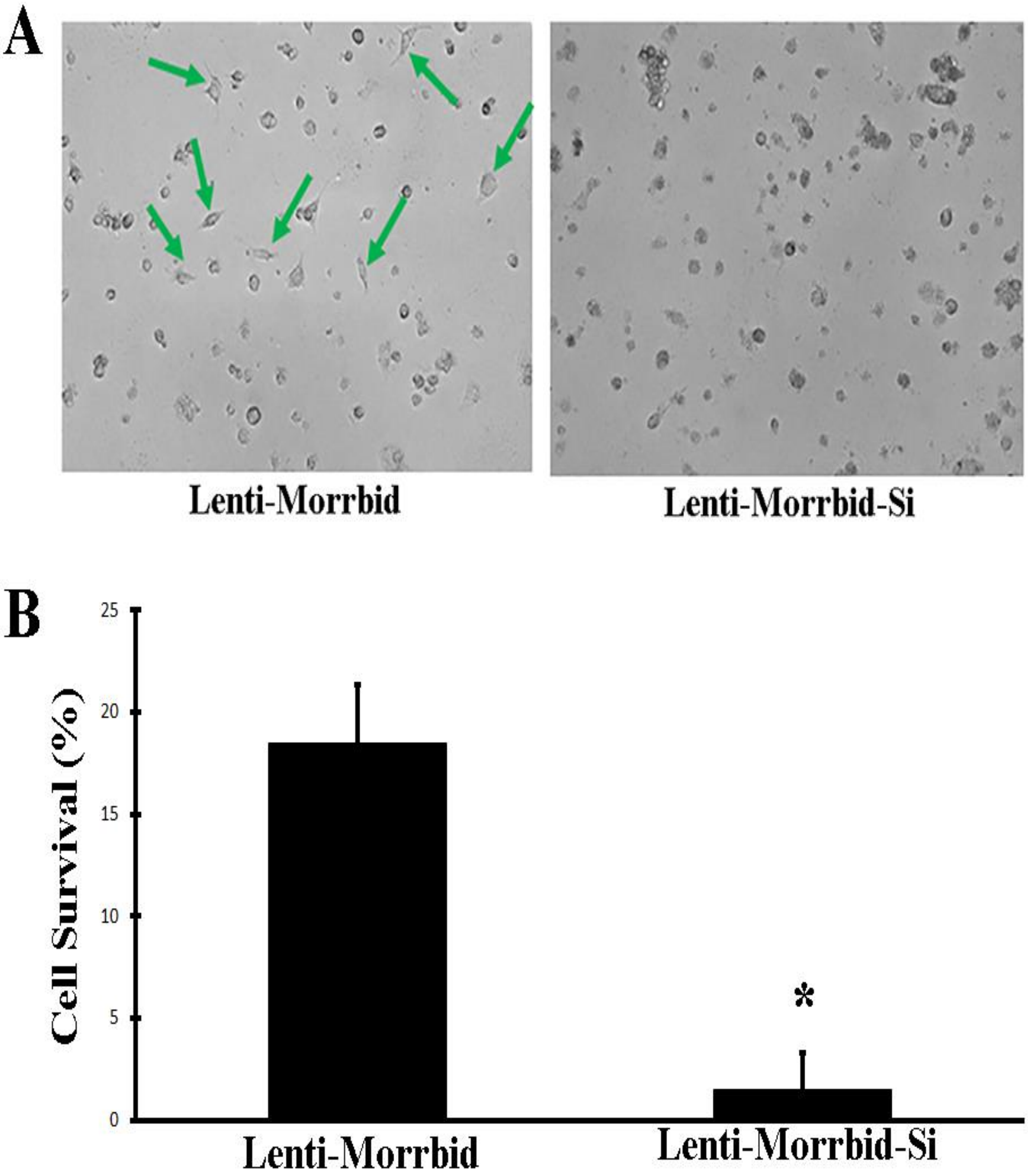

Supplemental Figure 9. The effect of *Morrbid* on monocyte survival. Overexpression of *Morrbid* could enhance, while Morrbid knockdown could reduce the monocyte survival. N=6; $p<0.05$ compared with lenti-Morrbid group,

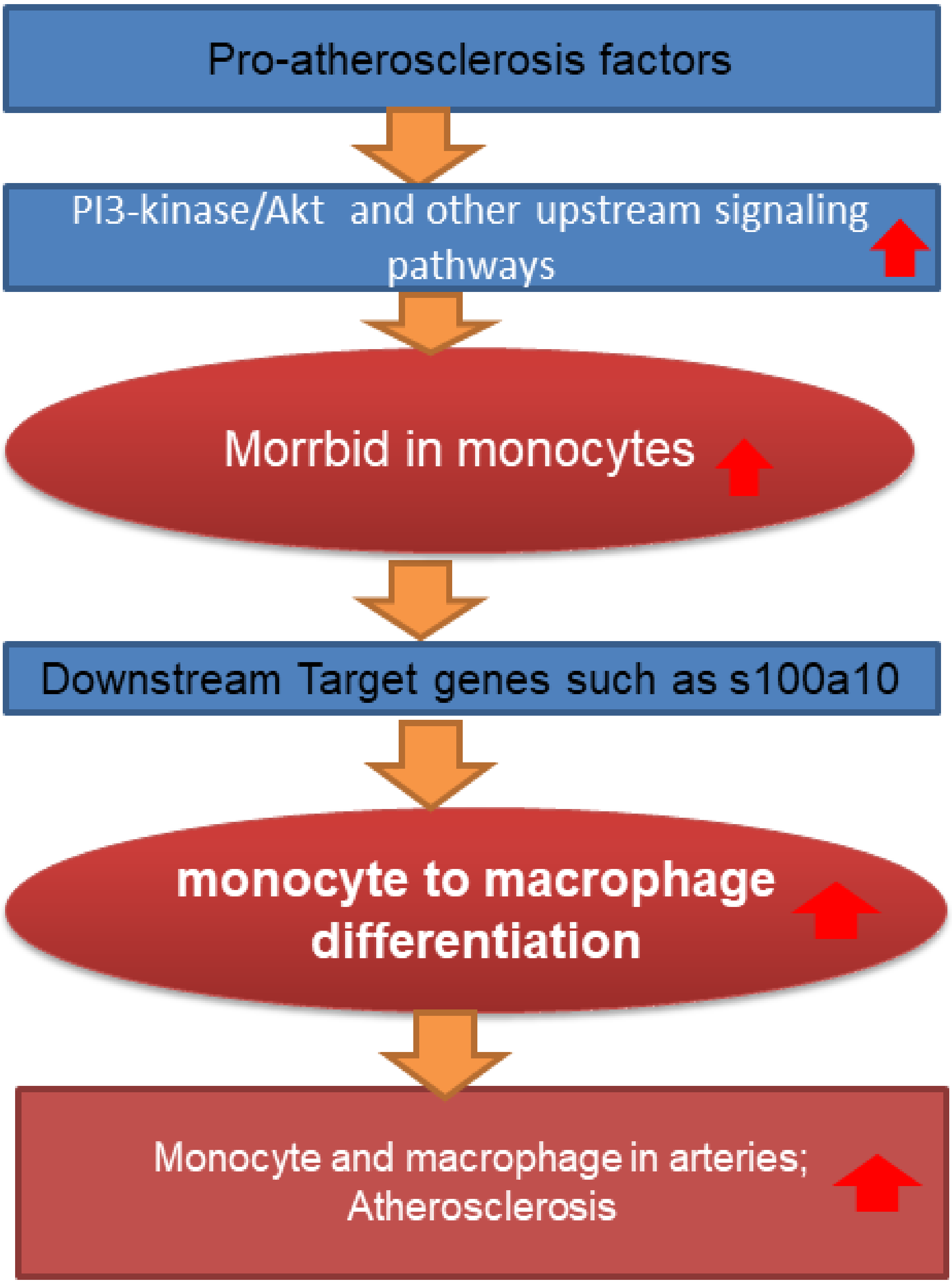

Supplemental Figure 10. A schematic overview of Morrbid in monocyte to macrophage differentiation and in atherogenesis, as well as the mechanisms involved.

Supplemental Tables

Table S1. Primers used in this study

Primer name	orientation	Primer sequences
GAPDH	Forward	TGCACCACCAACTGCTTAGC
	Reverse	GGCATGGACTGTGGTCATGAG
Human CD36	Forward	TCTTTCCTGCAGCCCAATG
	Reverse	AGCCTCTGTTCCAACTGATAGTGA
Human CD68	Forward	TCAGCTTTGGATTCATGCAG
	Reverse	AGGTGGACAGCTGGTGAAAG
Human CD71	Forward	TACTTAGCGGATGGTGATG
	Reverse	TTCTTGTTTGCCCTTTGC
EMR1	Forward	GATGAAGATCGGGTGTTCCACAA
	Reverse	CCATGCCCACAAAGGAGACAA
F4/80	Forward	CTTTGGCTATGGGCTTCCAGTC
	Reverse	GCAAGGAGGACAGAGTTTATCGTG
Mouse Morrbid	Forward	TCTGAGAATGAGGGGACTGG
	Reverse	TGTGCTGTGAAGATCCCAAG
Human Morrbid	Forward	ACTGGATGGTCGCTGCTTTT
	Reverse	CTTCCCAGGAACTGTGCTGT
EGE-ZY2-008-WT	Forward	ACTCTCTGGCACACATATCTGCTCT
	Reverse	AGCTGGCTGTTAGCATGTGTGACTT
EGE-ZY2-008-Mut	Forward	ACTCTCTGGCACACATATCTGCTCT
	Reverse	CCCATAGGAAACACAGGAGGCACTG
